# Hydrogen Permeability of Polyamide 6 Used as Liner Material for Type IV On-Board Hydrogen Storage Cylinders

**DOI:** 10.3390/polym15183715

**Published:** 2023-09-10

**Authors:** Chufeng Dong, Yitao Liu, Jiepu Li, Guangfu Bin, Chilou Zhou, Wulin Han, Xiang Li

**Affiliations:** 1 Hunan Provincial Key Laboratory of Health Maintenance for Mechanical Equipment, Hunan University of Science and Technology, Xiangtan 411201, China; 2 China Special Equipment Inspection and Research Institute, Beijing 100029, China; 3 Key Laboratory of Safety of Hydrogen Energy Storage and Transportation Equipment for State Market Regulation, Beijing 100029, China; 4 School of Mechanical and Automotive Engineering, South China University of Technology, Guangzhou 510641, China; 5 Hydrosys (Beijing) Technology Co., Ltd., Beijing 102627, China

**Keywords:** hydrogen storage cylinder, hydrogen permeability, diffusion, solubility

## Abstract

As a commonly used liner material for fully reinforced, carbon-fiber-composite hydrogen storage cylinders, polyamide 6 (PA6) needs to meet the required hydrogen permeation index during use; otherwise, it may adversely affect the safe use of hydrogen storage cylinders. The hydrogen permeability of PA6 under different temperatures and pressures was tested, and the variations in its hydrogen permeability were investigated. Additionally, the hydrogen permeability of PA6, polyamide 11 (PA11), and high-density polyethylene (HDPE) at a temperature of 288 K and a pressure of 70 MPa was tested, and the differences in hydrogen permeability among these commonly used liner materials for type IV on-board hydrogen storage cylinders were studied. The results reported herein indicate that both the hydrogen permeability and diffusion coefficient of PA6 increase with rising test temperature but decrease with increasing pressure. The solubility coefficient of PA6 shows no significant change with varying test temperatures and pressures. At a test temperature of 288 K and a pressure of 70 MPa, among the three materials, PA6 has slightly stronger hydrogen permeation resistance than PA11, while HDPE has the least resistance. These research findings can serve as valuable reference data for evaluating the hydrogen permeability of liner materials.

## 1. Introduction

As the main application of hydrogen energy in the field of transportation, hydrogen fuel cell vehicles have developed rapidly in recent years, and as one of the core components of hydrogen fuel cell vehicles, hydrogen storage cylinders have also been rapidly developed [1,2]. Currently, on-board hydrogen storage cylinders primarily consist of two types: type III and type IV. Of these, the type IV hydrogen storage cylinder has gained global attention due to its remarkable attributes, such as high hydrogen storage density, lightweight construction, and cost-effectiveness [3,4,5]. Internationally, nylon and high-density polyethylene are the predominant liner materials employed in type IV hydrogen storage cylinders. Under a high-pressure hydrogen environment, the polymer liner materials undergo hydrogen permeation behavior, and rapid pressure relief may cause damage such as liner buckling and blistering [6,7,8], which may affect the safe use of hydrogen storage cylinders.

The relevant standards [9,10,11] point out that hydrogen permeability is one of the key evaluation indexes in the study of the properties of hydrogen storage cylinder liner materials. The main factors affecting the hydrogen permeability of polymer materials are temperature, pressure and material properties [12,13,14,15,16]. Klopffer et al. [17,18] investigated the hydrogen permeability coefficients of PA11 and PA11 filled with ethylene vinyl alcohol (EVOH) used in hydrogen transport pipelines at various temperatures (303–363 K) and pressures (0.5–2 MPa). The results showed that the hydrogen permeability coefficient of PA11 filled with EVOH is reduced by 15–30 times compared to that of pure PA11, showcasing the effective enhancement of hydrogen permeation resistance achieved through the filler. The experiment only considered the hydrogen permeability of the material at relatively low pressures, and lacked exploration under high-pressure conditions. Pepin et al. [19] studied the hydrogen permeability coefficients of two nylon materials (PA6, PA12) at a test pressure of 18 MPa and a temperature of 328 K. Their findings indicated that PA12 exhibited a significantly higher hydrogen permeability coefficient compared to PA6. Humpenoder et al. [20] conducted a study on the permeability coefficient, diffusion coefficient, and solubility coefficient of PA materials in a hydrogen environment, but the test temperature range was limited to 273–303 K. Yamabe et al. [21] proposed the application of thermal desorption analysis (TDA) for measuring the hydrogen permeability coefficient of polymer materials. In a sealed container, the samples were exposed to a high-pressure hydrogen environment, and the amount of hydrogen passing through the polymer materials was measured using gas chromatography. Hydrogen permeation-related parameters were calculated through function fitting. Fujiwara et al. [22,23] developed a device capable of measuring the hydrogen permeability of materials under ultrahigh-pressure environments and evaluated the hydrogen permeability of HDPE using the high-pressure hydrogen gas permeation test (HPHP) method under various pressures (10–90 MPa) at a test temperature of 303 K. The results demonstrated that the hydrogen permeability of the material increased with the increase in pressure, but at the same time, the increase rate showed a gradually decreasing trend. Sun et al. [24] investigated the applicability of PA6 filled with layered inorganic components (LIC) as the liner material for hydrogen storage cylinders. They compared the hydrogen permeability of LIC/PA6 with that of PA6 under different test temperature and pressure conditions. Barth et al. [25] investigated the temperature dependency of hydrogen permeability of several polymer materials including HDPE, PA, PVC, and butyl rubber. The results revealed that the hydrogen permeability coefficients for all materials increased with rising temperature. In summary, the existing research on the hydrogen permeability of polymer materials, both domestically and internationally, has been largely confined to specific and limited test conditions. To comprehensively study the hydrogen permeability of liner materials under practical service conditions, there is a need to conduct research encompassing actual operational conditions. Obtaining hydrogen permeation data under such realistic circumstances will offer valuable insights and references for selecting the most suitable liner material.

In Section 2 of this research paper, we provide a comprehensive description of the sample preparation, test device, and test method utilized in the study. Subsequently, Section 3 entails the hydrogen permeability testing of PA6 under varying test temperatures (308, 328, 358 K) and pressures (15, 43.75, 87.5 MPa). The pertinent test data for PA6 are acquired, facilitating a rigorous analysis of its hydrogen permeability variation across diverse test conditions. Furthermore, we conduct hydrogen permeability tests for PA6, PA11, and HDPE at a constant temperature of 288 K and a pressure of 70 MPa, thereby elucidating the differences in hydrogen permeability among frequently employed liner materials for type IV on-board hydrogen storage cylinders. In Section 4, the findings are systematically organized and summarized.

## 2. Experimental

### 2.1. Sample Preparation

**Figure 1:**
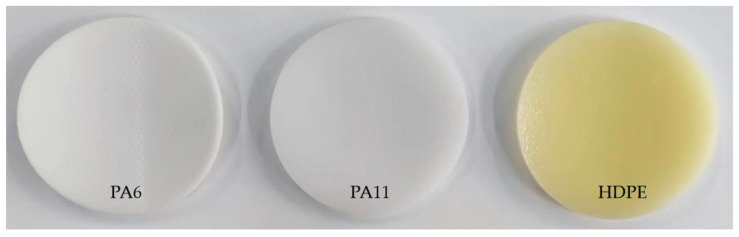
Hydrogen permeation test samples.

The PA6, PA11, and HDPE samples utilized in this study were sourced from a domestic manufacturer of type IV hydrogen storage cylinders. To ensure consistent test conditions, the hydrogen permeation test samples were prepared in accordance with GB/T 42610-2023 standards [26]. To prepare the samples for hydrogen permeation testing, sections were carefully excised from both sides of the injection-molded liner weld using an angular grinder. Subsequently, round samples, with a diameter of (78 ± 1) mm, essential for the hydrogen permeation tests, were precisely cut using a water-knife cutting machine, as depicted in Figure 1. It is noteworthy that the diameter of the sample and the area of contact with hydrogen should be equal to or greater than 25 mm, while the sample thickness aligns with the thickness of the liner. Surface cleaning of the specimens was performed using sandpaper before the initiation of the tests.

### 2.2. Hydrogen Permeation Test Device and Test Procedure

**Figure 2:**
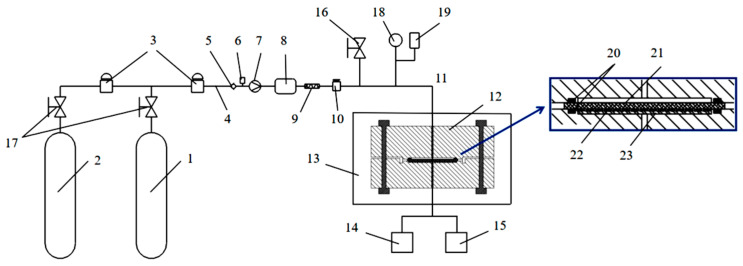
Schematic diagram of hydrogen permeation device. The parts indicated in the diagram are as follows: 1. Hydrogen cylinder group, 2. Nitrogen cylinder group, 3. Solenoid valve, 4. Gas supply piping, 5. Check valve, 6. Pressure sensor, 7. Booster pump, 8. Buffer tank, 9. Gas molecular filter, 10. Gas supply control valve, 11. Gas intake piping, 12. Permeation cell, 13. Environmental chamber, 14. Vacuum pump, 15. Vacuum gauge, 16. Venting valve, 17. Manual valve, 18. Pressure gauge, 19. Pressure sensor, 20. O-ring, 21. Sample, 22. Metal wire screen, 23. Sintered metal support plate.

The hydrogen permeation test device employed in this study is depicted in Figure 2, facilitating hydrogen permeation tests of polymer materials at operating pressures ranging from 0.1 to 99 MPa and temperatures between 233 and 373 K. The test procedure for the device is as follows: (1) Prior to commencing the test, O-rings are installed in both the high-pressure and low-pressure cavities. Subsequently, the sintered metal support plate, metal wire screen, and hydrogen permeation test sample are sequentially placed. (2) The high-pressure and low-pressure cavities are securely fastened with bolts, ensuring the proper installation and airtight sealing of the permeation device. (3) The environmental chamber’s temperature parameters are adjusted in accordance with the specified conditions for the hydrogen permeation test. Following a 2 h insulation period, the test is initiated. (4) The low-pressure side is subjected to vacuuming, while nitrogen purging and hydrogen replacement are performed for the high-pressure side pipeline and high-pressure cavity. (5) With the manual valve of the hydrogen cylinder opened, the hydrogen booster pump is activated to achieve pressurization of the high-pressure side to the designated test pressure. (6) Throughout the test, real-time pressure changes on the low-pressure side are monitored using a high-precision vacuum gauge. The test is concluded once the pressure change stabilizes.

To ensure proper cavity assembly, the sample’s diameter should be polished before assembly, and the support gasket’s thickness inside the tool must be adjusted according to the sample’s thickness. To prevent deformation and leakage of the round sample under high-pressure conditions, the hydrogen permeation test sample is placed over the metal wire screen and sintered metal support plate. This arrangement prevents excessive deformation caused by surface extrusion, and the selection of the sintered metal’s opening rate should ensure that the hydrogen permeation process is not affected. To ensure effective installation and sealing of the main body of the permeation device, and to prevent leakage on either the high- or low-pressure side of the sample, an O-ring static sealing structure is employed between the sample and the cavity on both sides. It is essential to replace the sealing ring for each hydrogen permeation test promptly. The control precision of the temperature control device in the environmental chamber is no less than ±1 K, and the accuracy of the pressure sensor in the experimental system is no less than ±1% of the test pressure. It has been verified that the accuracy of the test components has a negligible effect on the calculation results of hydrogen permeation-related parameters.

### 2.3. Test Method for Hydrogen Permeability of Materials

The hydrogen permeability coefficient of the material can be calculated from the following equation [27]:

(1) $$P_{e}=\frac{\Delta p}{\Delta t}\times \frac{V}{s}\times \frac{T_{0}}{P_{0}T}\times \frac{d}{\left(p_{1}-p_{2}\right)}$$

where *P_e_* is the gas permeability coefficient of the material (cm^3^·cm/(cm^2^·s·Pa)), Δ*p*/Δ*t* is the arithmetic mean of the change of gas pressure on the low pressure side per unit time when it is stable permeation (Pa/s), *V* is the volume of the low pressure chamber (cm^3^), *s* is the test area of the sample (cm^2^), *T*_0_ and *P*_0_ are the temperature (273.15 K) and the pressure (1.0133 × 10^5^ Pa) under the standard conditions, *T* is the test temperature (K), *d* is the thickness of the sample (cm), and *p*_1_ *− p*_2_ is the pressure difference between the two sides of the sample (Pa).

According to the time lag method, the diffusion coefficient of gas in the material is determined, also known as the “high vacuum method”, that is, under high vacuum, the diffusion coefficient *D* is calculated by the “lag time” when the equilibrium state is reached:

(2) $$D=\frac{l^{2}}{6\theta }$$

where *l* is the thickness of the sample (cm), *θ* is the lag time (s), and *D* is the diffusion coefficient (cm^2^/s).

The permeability coefficient *P_e_* is the product of the diffusion coefficient *D* and the solubility coefficient *S*. Therefore, the solubility coefficient of the gas in the material can be calculated by the following equation, and its unit is cm^3^/(cm^2^·s·cm·Hg).

(3) $$S=\frac{P_{e}}{D}$$

**Figure 3:**
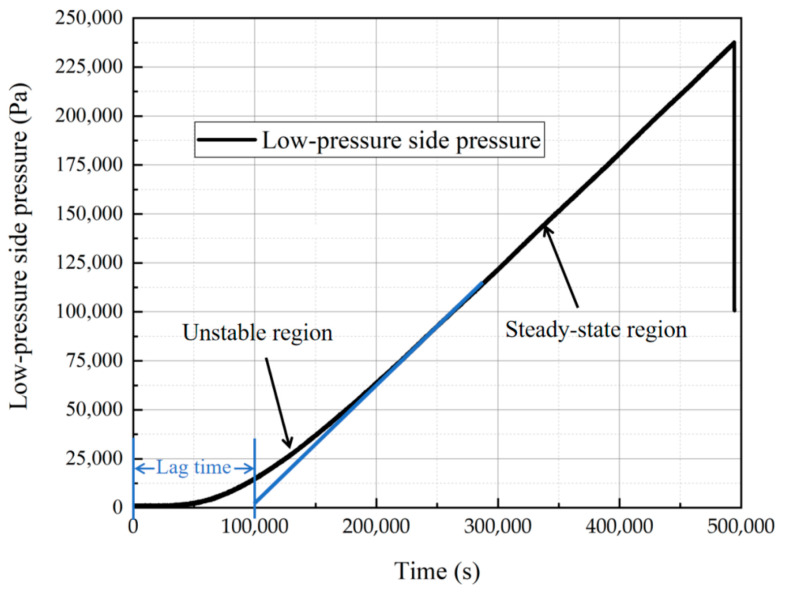
Pressure variation curve for the low-pressure side through time.

Taking the hydrogen permeation test of PA6 at the test temperature of 288 K and test pressure of 70 MPa as an example, this section provides calculations, explanations, and error analysis of key hydrogen permeation parameters. Figure 3 illustrates the pressure change on the low-pressure side during the hydrogen permeation test. The permeation process comprises an unstable region and a steady-state region. Sampling in the steady-state region was performed at least once every 30 min until the pressure deviations between any five consecutive measurements within a period exceeding 24 h did not exceed ±5%. When this criterion was met, indicating the stabilization in the low-pressure side pressure variations, the test could be concluded.

In the steady-state permeation region, five continuous segments of permeation data from equidistant time intervals are selected for calculating key hydrogen permeation parameters. The chosen time intervals should be neither excessively long nor too short to ensure the accuracy of parameter calculations. Trend predictions are conducted separately for the data from the five permeation segments, yielding linear functions. The slope of these functions represents Δ*p*/Δ*t*, while the intercept stands for the lag time. The volume of the low-pressure chamber is 7.8 cm³, the known permeation test area of the specimen is 12.566 cm², and the specimen thickness measures 0.35 cm. Based on the aforementioned equations, the hydrogen permeability coefficient, diffusion coefficient, and solubility coefficient of the PA6 specimen are calculated. The corresponding data are presented in Table A1. After excluding the maximum and minimum hydrogen permeability values among the five sets of data, the remaining three sets are averaged to derive the hydrogen permeability, diffusion coefficient, and solubility coefficient of PA6 at the experimental temperature of 288 K and test pressure of 70 MPa.

## 3. Results and Discussion

### 3.1. Effect of Different Test Temperatures and Pressures on the Hydrogen Permeability Coefficient of PA6

The liner material of type IV hydrogen storage cylinders is subjected to alternating pressure loads and continuous temperature changes under actual operating conditions, which could potentially affect the hydrogen permeability of liner materials. To investigate the effect of different test temperatures and pressures on the hydrogen permeability of PA6, hydrogen permeation tests were conducted on round samples of PA6 at varying temperatures (308, 328, 358 K) and pressures (15, 43.75, 87.5 MPa). The resulting hydrogen permeability coefficient of each sample is tabulated in Table 1.

**Figure 4:**
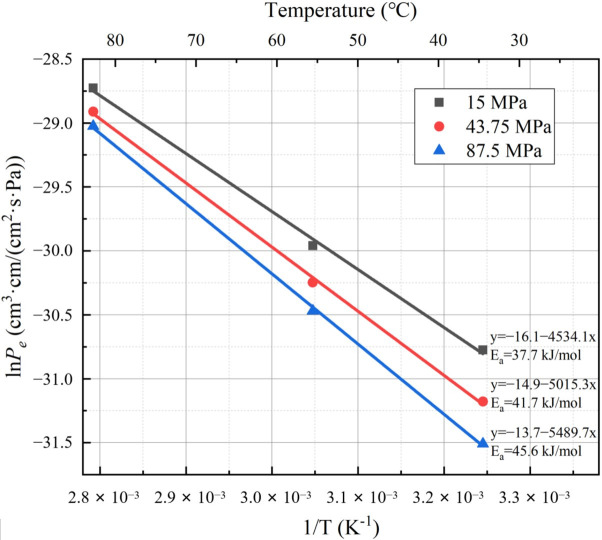
Fitting curve for the hydrogen permeability coefficient of PA6 with temperature under different test pressures.

**Figure 5:**
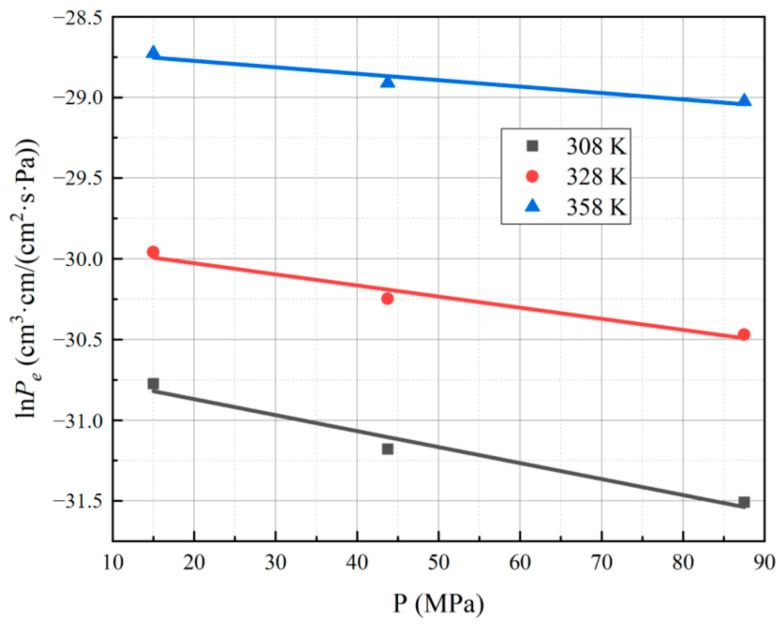
Fitting curve for the hydrogen permeability coefficient of PA6 with pressure under different test temperatures.

The results reveal that at a hydrogen permeation test temperature of 358 K and pressure of 15 MPa, PA6 exhibits the highest hydrogen permeability coefficient, measuring 3.34 × 10^−13^ cm^3^·cm/(cm^2^·s·Pa). Conversely, at a test temperature of 308 K and pressure of 87.5 MPa, PA6 displays the smallest hydrogen permeability coefficient, measuring 2.07 × 10^−14^ cm^3^·cm/(cm^2^·s·Pa). The data are plotted in Figure 4 and Figure 5, where the reciprocal of temperatures and pressures serve as the horizontal coordinates, respectively, and the logarithm of hydrogen permeability coefficients serve as the vertical coordinates.

The relationship between the hydrogen permeability coefficient and temperature of PA6 is shown in Figure 4; a clear linear relationship is observed between the logarithm of the hydrogen permeability coefficient for PA6 and the reciprocal of temperature. It is evident that the hydrogen permeability coefficient of PA6 increases with rising temperature. This observed variation aligns with Arrhenius’ law [26], indicating that the relationship between hydrogen permeability and temperature follows this kinetic theory. Concerning PA6, this result is consistent with the law of variation in hydrogen permeability coefficient with temperature obtained by Sun et al. [24] based on a hydrogen permeability test. Additionally, an assessment of the activation energy of PA6 materials was conducted at different experimental pressures (15, 43.75, 87.5 MPa), resulting in activation energies of approximately 37.7, 41.7, and 45.6 kJ/mol, respectively. This arises due to the impact exerted by varying pressures on the microstructural characteristics and internal motion dynamics of the material. By fitting the data obtained by Sun, the activation energies of PA6 were determined to be 21.4, 22.6, and 23.2 kJ/mol under 25, 35, and 50 MPa experimental pressures, respectively. This discrepancy may arise from differences in the material’s manufacturing processes, leading to distinct structural characteristics and significant variation in the activation energy of PA6. The hydrogen permeability of PA6 is jointly determined by the material’s free volume and the segmental flow in the material. An increase in the test temperature enhances the molecular motion of PA6 chains, creating larger free spaces within the material. This facilitates easier permeation of hydrogen molecules through PA6. Additionally, as the test temperature gradually rises, the chain segments within the PA6 material are released from the frozen state. This prompts side groups, branches, or links to initiate mutual movement, thereby further promoting the permeation of hydrogen molecules through the PA6 material [28,29]. This mechanism accounts for the observed temperature-dependent increase in the hydrogen permeability coefficient of PA6, elucidating the influence of temperature on the material’s permeation behavior.

The relationship between the hydrogen permeability coefficient and pressure for PA6 is shown in Figure 5; it is evident that there exists a linear relationship between the logarithm of the hydrogen permeability coefficient and the experimental pressure for the PA6 material. This is consistent with the gas permeation behavior observed by Naito et al. [30] for low-density polyethylene (LDPE) and polypropylene (PP) films at an experimental temperature of 298 K and pressures ranging from 0 to 100 atm. For insoluble gases such as hydrogen, the permeability coefficient decreases with the increase in test pressure; this phenomenon can be attributed to the compression and densification of PA6 caused by the elevated experimental pressure [29]. Consequently, the internal free volume within the PA6 material reduces, impeding the diffusion of hydrogen molecules to a greater extent, thereby leading to a decreasing trend in the hydrogen permeability coefficient.

### 3.2. Effect of Different Test Temperatures and Pressures on the Diffusion Coefficient of PA6

**Figure 6:**
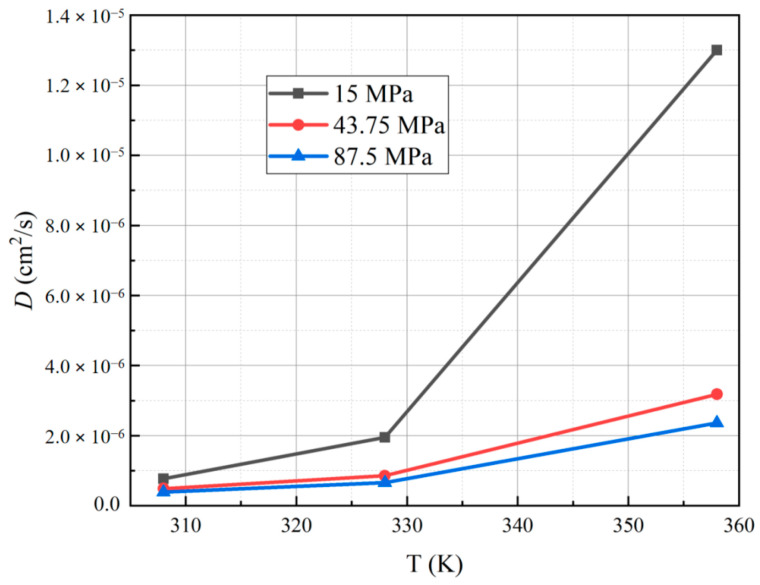
Curve for the diffusion coefficient of PA6 with temperature under different test pressures.

**Figure 7:**
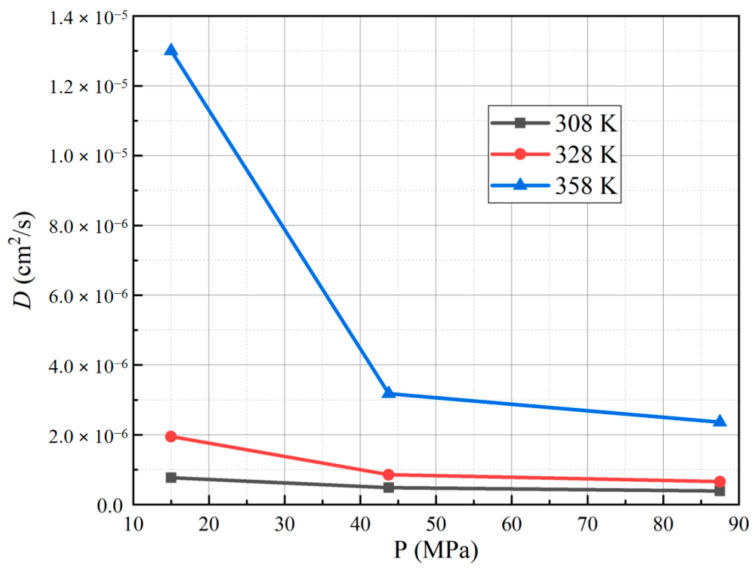
Curve for the diffusion coefficient of PA6 with pressure under different test temperatures.

Table 2 presents the diffusion coefficients obtained for each sample. The results indicate that, at a hydrogen permeation test temperature of 358 K and a pressure of 15 MPa, PA6 exhibits the highest diffusion coefficient, measuring 1.30 × 10^−5^ cm^2^/s. Conversely, at a test temperature of 308 K and a pressure of 87.5 MPa, PA6 displays the smallest diffusion coefficient, measuring 3.91 × 10^−7^ cm^2^/s. On the vertical axis, we report the diffusion coefficients as a function of temperatures and pressures, which are presented on the horizontal axes in Figure 6 and Figure 7, respectively.

From the diffusion coefficient versus temperature curve, it is clear that as the test temperature increases, the diffusion coefficient of PA6 also shows a corresponding increase. Furthermore, the lower the pressure, the more pronounced the increase in the diffusion coefficient. This phenomenon can be attributed to the energy required for hydrogen molecules to disassociate the intermolecular associations within the polymer matrix during their transfer. At elevated temperatures, the kinetic energy of the polymer chains is intensified, resulting in a higher mobility of hydrogen molecules within the polymer structure [22,24]. Consequently, the diffusion coefficient experiences a commensurate increment with the rise in temperature.

Based on the diffusion coefficient versus pressure curve, it is evident that the diffusion coefficient of PA6 exhibits a decreasing trend with the rise in test pressure. Fujiwara et al. [22,23] obtained the same results for HDPE at different test pressures (10–90 MPa). Moreover, the higher the temperature, the more pronounced the decrease in the diffusion coefficient. At higher test pressures, the free volume within the PA6 material decreases, hindering the diffusion of hydrogen within it. Additionally, Fumitoshi et al. [31] observed that under high-pressure hydrogen environments, the crystallinity of polymer materials increases, further impeding the diffusion of hydrogen. Therefore, as the test pressure increases, the material’s diffusion coefficient decreases.

### 3.3. Effect of Different Test Temperatures and Pressures on the Solubility Coefficient of PA6

**Figure 8:**
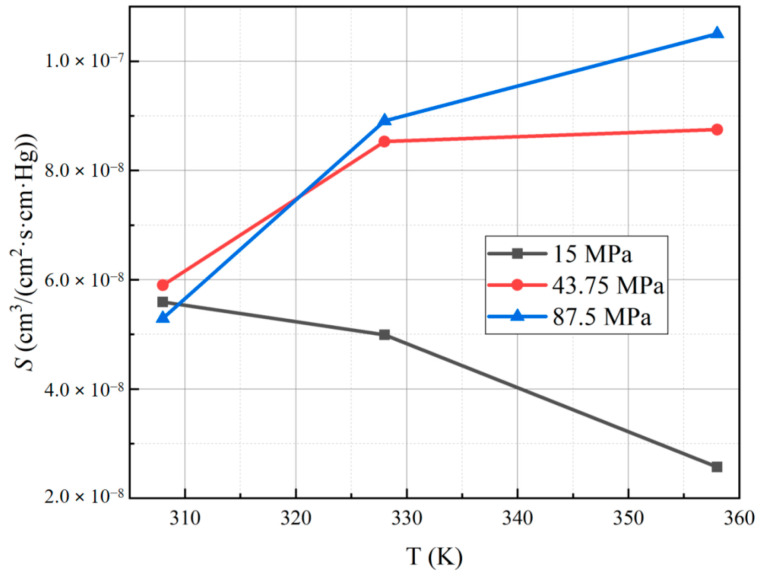
Curve for the solubility coefficient of PA6 with temperature under different test pressures.

**Figure 9:**
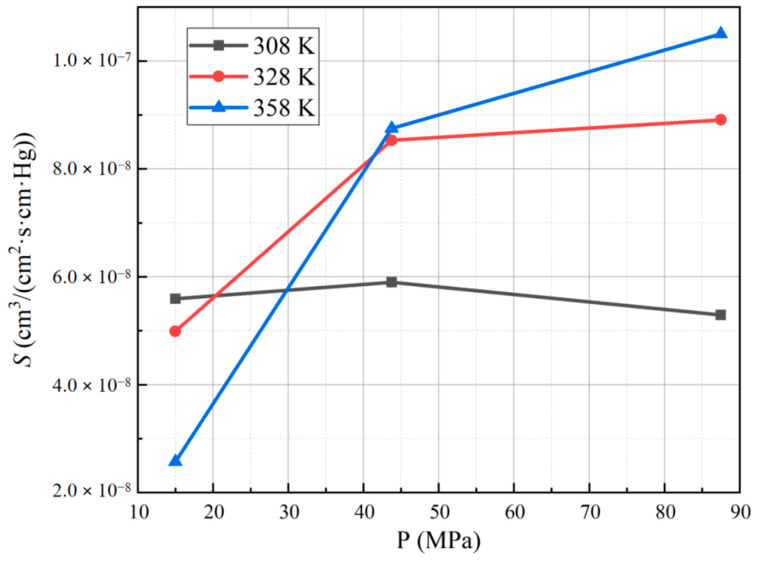
Curve for the solubility coefficient of PA6 with pressure under different test temperatures.

Table 3 presents the solubility coefficients obtained for each sample. The results indicate that at a hydrogen permeation test temperature of 358 K and a pressure of 87.5 MPa, PA6 exhibits the highest solubility coefficient, measuring 1.05 × 10^−7^ cm^3^/(cm^2^·s·cm·Hg). Conversely, at a test temperature of 358 K and a pressure of 15 MPa, PA6 displays the smallest solubility coefficient, measuring 2.57 × 10^−8^ cm^3^/(cm^2^·s·cm·Hg). On the vertical axis, we report the solubility coefficients as a function of temperature and pressure, which are presented on the horizontal axes in Figure 8 and Figure 9, respectively.

The solubility coefficient of PA6 does not change significantly with the test temperature and pressure. Typically, an increase in temperature leads to a reduction in gas solubility. However, in the case of hydrogen in PA6, the gas activity capacity increases with rising temperature, resulting in greater accumulation of hydrogen molecules within the material per unit time. Consequently, the amount of hydrogen dissolved inside the material increases, and the interplay of these two processes influences the solubility coefficient [15]. In summary, the solubility behavior of hydrogen in PA6 is characterized by a complex interplay between temperature and gas activity capacity, leading to a limited effect of test temperature and pressure on the solubility coefficient.

### 3.4. Differences in Hydrogen Permeability among Commonly Used Liner Materials for Type IV On-Board Hydrogen Storage Cylinders

To investigate the differences in hydrogen permeability among commonly utilized liner materials for type IV on-board hydrogen storage cylinders, we conducted hydrogen permeability tests on PA6, PA11, and HDPE at a temperature of 288 K and a pressure of 70 MPa. The hydrogen permeability coefficients, diffusion coefficients, and solubility coefficients of these distinct materials are calculated and presented in Table 4.

At a hydrogen permeation test temperature of 288 K and a pressure of 70 MPa, the hydrogen permeability coefficients of PA6, PA11, and HDPE are found to be 1.72 × 10^−14^, 1.87 × 10^−14^, and 5.88 × 10^−14^ cm^3^·cm/(cm^2^·s·Pa), respectively. In comparison to PA6, the hydrogen permeability coefficients of PA11 and HDPE experience an approximately 8.7% and 242% increase, respectively. The diffusion coefficients of PA6, PA11, and HDPE are determined to be 2.19 × 10^−7^, 2.43 × 10^−7^, and 9.73 × 10^−7^ cm^2^/s, respectively. Relative to PA6, the diffusion coefficients of PA11 and HDPE experience an approximately 12.5% and 350% increase, respectively. As for the solubility coefficients, the values for PA6, PA11, and HDPE are found to be 7.85 × 10^−8^, 7.71 × 10^−8^, and 6.05 × 10^−8^ cm^3^/(cm^2^·s·cm·Hg), respectively. Notably, the solubility coefficients of the three materials do not exhibit significant differences. Overall, the results indicate that PA6 demonstrates slightly stronger hydrogen permeation resistance than PA11, while HDPE exhibits the lowest hydrogen permeation resistance among the tested materials.

## 4. Conclusions and Prospect

In this study, hydrogen permeability of PA6 was tested under various test temperatures (308, 328, 358 K) and pressures (15, 43.75, 87.5 MPa). Additionally, hydrogen permeation tests were conducted on PA6, PA11, and HDPE materials at a temperature of 288 K and a pressure of 70 MPa. The following conclusions are drawn:

(1) The logarithm of the hydrogen permeability coefficient for PA6 demonstrates a linear relationship with the reciprocal of temperature, and the hydrogen permeability coefficient of the material increases with higher test temperatures. Additionally, the logarithm of the hydrogen permeability coefficient for PA6 exhibits a linear relationship with the test pressure, and the hydrogen permeability coefficient of the material decreases with higher test pressures.
(2) The diffusion coefficient of PA6 increases with higher test temperatures, and the lower the pressure, the more pronounced the increase in the diffusion coefficient. Additionally, the diffusion coefficient of PA6 decreases with higher test pressures, and the higher the temperature, the more pronounced the decrease in the diffusion coefficient. Notably, the solubility coefficient of PA6 does not show a significant dependence on the test temperature and pressure.
(3) At a test temperature of 288 K and a pressure of 70 MPa, it is observed that compared to PA6, the hydrogen permeability coefficients of PA11 and HDPE increase by approximately 8.7% and 242%, respectively. Similarly, the diffusion coefficients of PA11 and HDPE increase by about 12.5% and 350%, respectively, relative to PA6. On the other hand, the solubility coefficients of the three materials do not exhibit significant differences. Among the three materials, PA6 has slightly stronger hydrogen permeation resistance than PA11, while HDPE has the least resistance.

This article supplements the hydrogen permeation test data of the liner materials for type IV on-board hydrogen storage cylinders, which can provide references for the evaluation of hydrogen permeability of liner materials. However, this study focuses on hydrogen permeation tests of materials at room temperature and elevated temperatures, while hydrogen permeation tests in low-temperature environments have not been considered. Microcracks in polymer materials are more prone to expand under the combined effect of low temperature and load, which may result in a decrease in the hydrogen permeability of materials and even lead to low-temperature failure. Therefore, exploring whether the hydrogen permeation law of liner materials under low-temperature conditions is the same as that of the currently known material hydrogen permeation law is a key aspect of our future work.

## Figures and Tables

**Table 1:** Hydrogen permeability coefficient *P_e_* (cm^3^·cm/(cm^2^·s·Pa)) of PA6 at different test temperatures and pressures.

Conditions	*P_e_*	In*P_e_*
308 K, 15 MPa	4.31 × 10^−14^	−30.78
308 K, 43.75 MPa	2.88 × 10^−14^	−31.18
308 K, 87.5 MPa	2.07 × 10^−14^	−31.51
328 K, 15 MPa	9.75 × 10^−14^	−29.96
328 K, 43.75 MPa	7.31 × 10^−14^	−30.25
328 K, 87.5 MPa	5.85 × 10^−14^	−30.47
358 K, 15 MPa	3.34 × 10^−13^	−28.73
358 K, 43.75 MPa	2.78 × 10^−13^	−28.91
358 K, 87.5 MPa	2.48 × 10^−13^	−29.03

**Table 2:** Diffusion coefficient *D* (cm^2^/s) of PA6 at different test temperatures and pressures.

	Temperature	308 K	328 K	358 K
Pressure				
15 MPa		7.71 × 10^−7^	1.95 × 10^−6^	1.30 × 10^−5^
43.75 MPa		4.87 × 10^−7^	8.57 × 10^−7^	3.18 × 10^−6^
87.5 MPa		3.91 × 10^−7^	6.57 × 10^−7^	2.36 × 10^−6^

**Table 3:** Solubility coefficient *S* (cm^3^/(cm^2^·s·cm·Hg)) of PA6 at different test temperatures and pressures.

	Temperature	308 K	328 K	358 K
Pressure				
15 MPa		5.59 × 10^−8^	4.99 × 10^−8^	2.57 × 10^−8^
43.75 MPa		5.90 × 10^−8^	8.53 × 10^−8^	8.75 × 10^−8^
87.5 MPa		5.29 × 10^−8^	8.91 × 10^−8^	1.05 × 10^−7^

**Table 4:** Hydrogen permeation test results of different materials.

Materials	P_e_ (cm^3^·cm/(cm^2^·s·Pa))	D (cm^2^/s)	S (cm^3^/(cm^2^·s·cm·Hg))
PA6	1.72 × 10^−14^	2.19 × 10^−7^	7.85 × 10^−8^
PA11	1.87 × 10^−14^	2.43 × 10^−7^	7.71 × 10^−8^
HDPE	5.88 × 10^−14^	9.73 × 10^−7^	6.05 × 10^−8^

## Data Availability

Data are contained within the article.

## Appendix A

Taking the hydrogen permeation test of PA6 at the test temperature of 288 K and test pressure of 70 MPa as an example, the relevant test data can be found in Table A1.

**Table A1:** Hydrogen permeation test data for PA6 at a test temperature of 288 K and test pressure of 70 MPa.

Data Integration Diagram of Different Time Periods	Δ*p*/Δ*t* (pa/s)	*d* (cm)	Lag Time (s)	*P_e_* (cm^3^·cm/(cm^2^·s·Pa))	*D* (cm^2^/s)	*S* (cm^3^/(cm^2^·s·cm·Hg))
(a) 325,001–350,000 s	0.5903	0.35	93,294.93	1.714 × 10^−14^	2.188 × 10^−7^	7.832 × 10^−8^
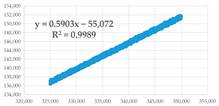
(b) 350,001–375,000 s	0.5861	0.35	91,585.05	1.701 × 10^−14^	2.229 × 10^−7^	7.634 × 10^−8^
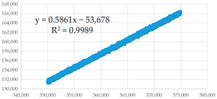
(c) 375,001–400,000 s	0.594	0.35	95,506.73	1.725 × 10^−14^	2.138 × 10^−7^	8.068 × 10^−8^
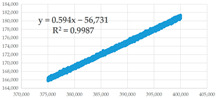
(d) 400,001–425,000 s	0.605	0.35	100,770.25	1.757 × 10^−14^	2.026 × 10^−7^	8.670 × 10^−8^
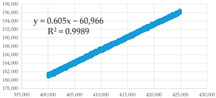
(e) 425,001–450,000 s	0.5884	0.35	91,578.86	1.708 × 10^−14^	2.229 × 10^−7^	7.663 × 10^−8^
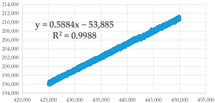
Average value	/	/	/	1.72 × 10^−14^	2.19 × 10^−7^	7.85 × 10^−8^

Remove the maximum and minimum hydrogen permeability coefficient values from among the five sets of data, that is, remove the sets (b) and (d), and average the relevant data for the remaining three groups, so as to obtain for PA6 the hydrogen permeability coefficient, diffusion coefficient, and solubility coefficient at the test temperature of 288 K and test pressure of 70 MPa. The calculation results are reported using two decimal places. The error between the calculated results of test data and the calculated mean of the hydrogen permeability coefficient, diffusion coefficient, and solubility coefficient is within ±3%, which can be considered insignificant.
